# Circ-MFN2 Positively Regulates the Proliferation, Metastasis, and Radioresistance of Colorectal Cancer by Regulating the miR-574-3p/IGF1R Signaling Axis

**DOI:** 10.3389/fgene.2021.671337

**Published:** 2021-05-19

**Authors:** Defeng Liu, Shihao Peng, Yangyang Li, Tao Guo

**Affiliations:** Department of General Surgery, The Fourth Affiliated Hospital of Anhui Medical University, Hefei, China

**Keywords:** CRC, circ-MFN2, miR-574-3p, IGF1R, progression

## Abstract

Numerous studies have shown that the expression of circular RNA (circRNA) is closely related to the malignant progression of cancer. However, the role of circ-MFN2 in colorectal cancer (CRC) is unclear. Our study aims to explore the role and mechanism of circ-MFN2 in CRC progression. The relative expression levels of circ-MFN2, microRNA (miR)-574-3p and insulin-like growth factor 1 receptor (IGF1R) were detected by quantitative real-time polymerase chain reaction (qRT-PCR). Cell viability was determined using 3-(4, 5-dimethyl-2 thiazolyl)-2, 5-diphenyl-2-H-tetrazolium bromide (MTT) assay. The colony number and radioresistance of cells were assessed using colony formation assay. Moreover, the migration and invasion of cells were measured using transwell assay. Tumor xenograft model was constructed to evaluate the effect of circ-MFN2 knockdown on CRC tumor growth. Furthermore, dual-luciferase reporter assay was used to verify the interaction between miR-574-3p and circ-MFN2 or IGF1R. In addition, the protein level of IGF1R was evaluated by western blot (WB) analysis. Circ-MFN2 expression was elevated in CRC tissues and cells. Knockdown of circ-MFN2 restrained the proliferation, migration, invasion, and radioresistance of CRC cells *in vitro*. Furthermore, silenced circ-MFN2 also reduced the tumor volume and weight of CRC *in vivo*. MiR-574-3p could be sponged by circ-MFN2, and its inhibitor reversed the suppression effect of circ-MFN2 silencing on CRC progression. Moreover, IGF1R was a target of miR-574-3p, and its overexpression reversed the inhibition effect of miR-574-3p mimic on CRC progression. In addition, circ-MFN2 could positively regulate IGF1R expression by sponging miR-574-3p. Our results revealed that circ-MFN2 promoted the proliferation, metastasis and radioresistance of CRC through regulating the miR-574-3p/IGF1R axis, suggesting that circ-MFN2 might be a novel therapeutic biomarker for CRC.

## Introduction

Colorectal cancer (CRC) is a common malignant tumor, which not only affects the digestive system, but also metastasizes to the lymph, liver and kidney (Arnold et al., 2017; Siegel et al., 2018). At present, the treatment of CRC is mainly surgery, supplemented by chemoradiotherapy (Mishra et al., 2013). In particular, the use of immune checkpoint inhibitors is highly beneficial for immunotherapy in CRC with mismatch repair defect (dMMR) or microsatellite instability high (MSI-H) (Sahin et al., 2019; Schrock et al., 2019). However, the occurrence of tumor metastasis and radioresistance seriously affect the prognosis of CRC patients and markedly increase the treatment difficulty of CRC (Hohla et al., 2014; Lee and Oh, 2016). Therefore, understanding the molecular mechanisms that affect the metastasis and radioresistance of CRC are critical to improve the treatment strategies of CRC.

Circular RNA (circRNA) is a special non-coding RNA molecule newly discovered in recent years. Compared with traditional linear RNA, circRNA has a closed-loop structure and more stable expression (Ebbesen et al., 2017; Belousova et al., 2018). Functionally, circRNA has been found to have many microRNA (miRNA) binding sites and can act as miRNA sponge, which called the competitive endogenous RNA (ceRNA) mechanism of circRNA (Thomson and Dinger, 2016; Witkos et al., 2018). Researches have indicated that circRNA expression is closely related to cancer progression, including CRC (Taborda et al., 2017; Cui et al., 2018). For example, hsa_circ_0053277 was upregulated in CRC and could promote CRC proliferation and metastasis by regulating the miR-2467-3p/MMP14 network (Xiao and Liu, 2020). Knockdown of hsa_circ_0001313 could inhibit the radioresistance of colon cancer by sponging miR-338-3p (Wang L. et al., 2019).

Through the GEO database, we screened the differentially expressed circRNA in CRC tumor tissues and non-cancer tissues, and found that hsa_circRNA_100053 (circ-MFN2, also known as circ_0009910) was significantly upregulated in CRC tumor tissues. Nevertheless, the role of circ-MFN2 in CRC progression has not been studied. Circ-MFN2 has been reported to be highly expressed in osteosarcoma, hepatocellular carcinoma, and gastric cancer, and can promote the development of cancer (Deng et al., 2018; Liu et al., 2018; Li and Liu, 2020). Therefore, we speculated that circ-MFN2 might also play an active role in CRC. Here, we explored the role of circ-MFN2 in the proliferation, metastasis and radioresistance of CRC and investigated the underlying mechanism of circ-MFN2, hoping to provide new potential targets for CRC treatment.

## Materials and Methods

### Tissue Collection

CRC tissues and non-cancer tissues were obtained from 50 CRC patients who underwent surgical resection in The Fourth Affiliated Hospital of Anhui Medical University. The clinicopathologic features of CRC patients were shown in Table 1. CRC tissues were classified according to different TNM stages (I–II: *n* = 28; III–IV: *n* = 22) and whether lymph node metastasis occurred (Yes: *n* = 32; No: *n* = 18). All tissues were stored at –80°C. This study was approved by the Ethics Committee of The Fourth Affiliated Hospital of Anhui Medical University and obtained informed consent from all patients.

**TABLE 1:** Relationship between circ-MFN2 expression and clinicopathologic features of colorectal cancer patients.

		circ-MFN2 expression			
		
	Characteristics *n* = 50	Low (*n* = 16)	Medium (*n* = 17)	High (*n* = 17)	*P*-value^a^
Gender					0.6911
Female	21	8	7	6	
Male	29	8	10	11	
Age (years)					0.6904
≤60	18	7	6	5	
>60	32	9	11	12	
TNMgrade					0.011*
I + II	28	13	10	5	
III + IV	22	3	7	12	
Lymph node metastasis					0.010*
Positive	32	6	11	15	
Negative	18	10	6	2	
Tumor size					0.0033*
≤3 cm	19	11	6	2	
>3 cm	31	5	11	15	

TNM, tumor-node-metas-tasis; *P < 0.05.

*^*a*^Chi-square test.*

### Cell Culture and Transfection

CRC cell lines (LOVO, HCT-116, SW620 and SW480) were obtained from American Type Culture Collection (ATCC, Manassas, VA, United States) and normal intestinal epithelial cell line (NCM460) was purchased from EK-Bioscience (Shanghai, China). All cells were cultured in RPMI-1640 medium (Gibco, Grand Island, NY, United States) supplemented with 10% fetal bovine serum (FBS; Gibco) and 1% penicillin/streptomycin (Gibco) at 37°C with 5% CO_2_. Cell transfection was performed when the cells reached 60% confluences. Circ-MFN2 small interference RNA, lentiviral short hairpin RNA and pcDNA overexpression plasmid (si-circ-MFN2, sh-circ-MFN2 and circ-MFN2) or their controls (si-NC, sh-NC, and pcDNA), miR-574-3p mimic and inhibitor (miR-574-3p and anti-miR-574-3p) or their controls (miR-NC and anti-miR-NC), pcDNA insulin-like growth factor 1 receptor (IGF1R) overexpression plasmid and its control (pcDNA) were obtained from General Biosystems (Anhui, China). Lipofectamine 3000 (Invitrogen, Carlsbad, CA, United States) was used as the transfection reagent in this experiment.

### Quantitative Real-Time Polymerase Chain Reaction (qRT-PCR)

RNX-Plus kit (CinnaGen, Tehran, Iran) was used for extracting RNA, and cDNA Synthesis SuperMix kit (gDNA digester plus) (Yeasen, Shanghai. China) was used for synthesizing cDNA. QRT-PCR reaction was done using SYBR Green (Invitrogen). Relative expression was quantified using the 2^–ΔΔ*Ct*^ method and normalized by GAPDH or U6. All primer sequences were shown in Table 2.

**TABLE 2:** The primer sequences used for qRT-PCR.

Gene	Forward sequence (5′–3′)	Reverse sequence (5′–3′)
circ-MFN2	AGAGGCATCAGTGAGGTGCT	AAGTGCTTAAGTGGGGATGC
MFN2	GACCCCGTTACCACAGAAGA	GCAGAACTTTGTCCCAGAGC
miR-574-3p	GCCGAGCACGCTCATGCACACA	CTCAACTGGTGTCGTGGA
IGF1R	AACCCCAAGACTGAGGTGTG	TGACATCTCTCCGCTTCCTT
GAPDH	AAGGCTGTGGGCAAGGTCATC	GCGTCAAAGGTGGAGGAGTGG
U6	CTCGCTTCGGCAGCACA	AACGCTTCACGAATTTGCGT

### Ribonuclease R (RNase R) Assay

After extracted RNA from SW620 and SW480 cells, the RNA (20 μg) was incubated with RNase R (3 U/μg; Epicentre, Madison, WI, United States) for 30 min at 37°C. Then, the expression levels of circ-MFN2 and linear MFN2 were examined by qRT-PCR.

### 3-(4, 5-dimethyl-2 thiazolyl)-2, 5-diphenyl-2-H-tetrazolium Bromide (MTT) Assay

Using MTT Kit (Trevigen, Gaithersburg, MD, United States), cell viability was measured. Briefly, transfected and non-transfected SW480 and SW620 cells were seeded into 96-well plates (4 × 10^3^ cells/well). After 24, 48, and 72 h, MTT solution was added to cells and hatched for 4 h. Then, detergent reagent was added to solubilize the formazan. Optical density (OD) value was measured at 490 nm using a microplate reader to assess cell viability.

### Colony Formation Assay

This assay was used for measuring the colony number and radioresistance of cells. Transfected and non-transfected SW480 and SW620 cells were seeded into 6-well plates. For detecting cell colony number, the cells were cultured for 2 weeks, and then the colonies were fixed with methanol and stained with crystal violet. The number of colonies (> 50 cells) was counted under a microscope. For evaluating cell radioresistance, the cells were irradiated with X-ray at different radiation doses (0, 2, 4, 6, and 8 Gy). After 2 weeks, cells were fixed and stained, and the colony number was counted to calculate the survival fraction of cells.

### Transwell Assay

Transwell chambers (BD Biosciences, Franklin Lakes, NJ, United States) and Matrigel-coated transwell chambers (BD Biosciences) were used for detecting cell migration and invasion, respectively. SW480 and SW620 cells were seeded in the upper chamber with serum-free medium, and serum medium was added to the lower chamber. After 24 h, the cells on the surface of lower chambers were fixed and stained, and cell number was counted under a microscope (100×).

### Tumor Xenograft Model

SW480 cells (4 × 10^6^) transfected with sh-circ-MFN2 or sh-NC were subcutaneously injected into the right back of the nude mice (Hunan SJA Laboratory Animal Co., Ltd., Hunan, China). Each group was divided into 2 groups (*N* = 8). One group was given to 6 Gy X-ray irradiation (IR) after 8 days, and the tumor length and width of each group were detected. After that, the mice were irradiated and the tumor volume was measured every 4 days. After 31 days, the tumor growth curve was plotted and the tumor was removed for weighting and detecting circ-MFN2 expression. Mice were killed by cervical dislocation after deep anesthesia with 2% isoflurane. Animal experiments were performed in The Fourth Affiliated Hospital of Anhui Medical University and approved by the Animal Research Committee of The Fourth Affiliated Hospital of Anhui Medical University.

### Dual-Luciferase Reporter Assay

The segments of circ-MFN2 containing predicted binding sites or mutated binding sites of miR-574-3p were amplified and inserted into the psiCHECK-2 vector (Biovector, Beijing, China) to yield the wild-type (WT) or mutant-type (MUT) circ-MFN2 vector (WT/MUT-circ-MFN2). Then, the IGF1R 3′UTR-WT/MUT vector was also built in the same way. SW480 and SW620 cells were co-transfected with the above vectors and miR-574-3p mimic or miR-NC using Lipofectamine 3000. After incubation for 48 h, the luciferase activity was detected by Dual-Lucy Assay Kit (Solarbio, Beijing, China).

### Western Blot (WB) Analysis

Total protein was extracted from tissues and cells using RIPA Lysis Buffer (Solarbio) and quantified using BCA Kit (Solarbio). Protein was resolved by 10% SDS-PAGE gel (Beyotime, Shanghai, China) and then transferred onto PVDF membranes (Membrane Solutions, Nantong, China). After blocked with 5% non-fat milk, the membranes were hatched with primary antibodies against IGF1R (1:2,000, Bioss, Beijing, China), MFN2 (1:1,000, Bioss) or GAPDH (1:10,000, Bioss). The membranes were then hatched with secondary antibody (1:10,000, Bioss), and the signals were detected using enhanced chemiluminescence solution (Yeasen). Protein results was quantified using GAPDH.

### Statistical Analysis

All the experiments were performed in triplicate. GraphPad Prism 6.0 (GraphPad, La Jolla, CA, United States) was used for data analysis. Experimental data were presented as mean ± standard deviation, and statistical analysis was carried out using Student’s *t*-test or one-way analyses of variance followed by Tukey *post hoc* analysis. Log-rank test was used for Kaplan-Meier analysis and correlation analysis was performed using Pearson correlation analysis. *P* < 0.05 was considered significant.

## Results

### Upregulation of circ-MFN2 Was Found in CRC Tissues and Cells

**FIGURE 1:**
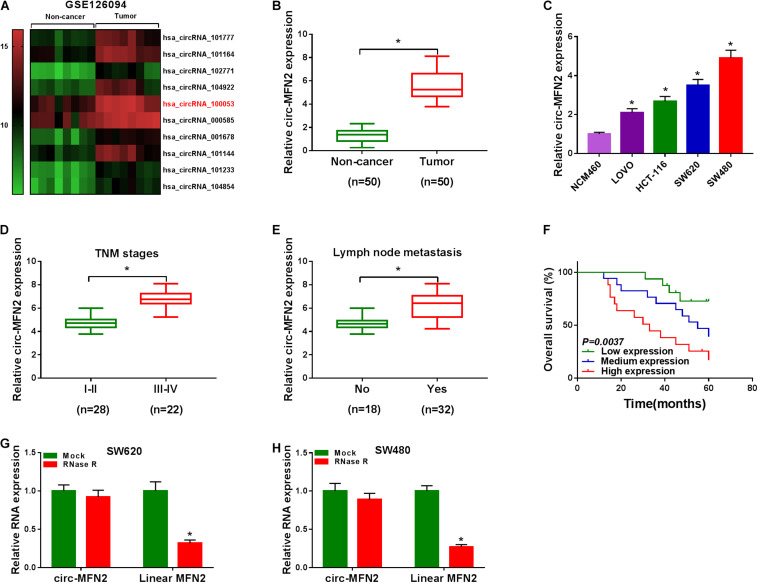
The expression of circ-MFN2 in CRC tissues and cells. **(A)** Heat map revealed that the differentially expressed circRNAs in 8 paired CRC tumor tissues and non-cancer tissues (GEO accession: GSE126094). **(B)** The expression of circ-MFN2 in CRC tumor tissues and non-cancer tissues was measured using qRT-PCR. **(C)** QRT-PCR was used to detect the circ-MFN2 expression in CRC cell lines (LOVO, HCT-116, SW620 and SW480) and NCM460 cells. **(D)** The expression of circ-MFN2 in different TNM stages (I-II and III-IV) of CRC patients was determined using qRT-PCR. **(E)** The expression of circ-MFN2 was detected by qRT-PCR in CRC patients with (Yes) or without (No) lymph node metastasis. **(F)** Kaplan-Meier analysis was performed to analyze the relationship between the circ-MFN2 expression and the overall survival rate of CRC patients. **(G,H)** RNase R assay was used to confirm the circular structure of circ-MFN2 compared to linear MFN2. *P < 0.05.

According to the cut-off criteria (log2 | fold change| > 1 and *P* < 0.05), we identified 10 differentially expressed circRNAs in 8 paired CRC tumor tissues and non-cancer tissues (GEO accession: GSE126094) (Figure 1A). The circRNA, hsa_circRNA_100053 (circ-MFN2), with the highest expression difference was selected for this study. We detected the expression of circ-MFN2 in CRC tumor tissues and found that circ-MFN2 was markedly highly expressed in CRC tumor tissues compared to non-cancer tissues (Figure 1B). Besides, in four CRC cell lines (LOVO, HCT-117, SW620, and SW480), we also discovered the increased expression of circ-MFN2 compared with that in NCM460 cells (Figure 1C). Furthermore, circ-MFN2 was also significantly upregulated in the tissues of the advanced stage of CRC patients (III-IV) and those with lymph node metastasis (Yes) (Figures 1D,E). Through analysis, we found that the high expression of circ-MFN2 was associated with TNM stage, lymph node metastasis and tumor size (Table 1). In addition, Kaplan-Meier analysis showed that high circ-MFN2 expression was often associated with the lower overall survival rate of CRC patients (Figure 1F). Using the RNase R assay, we found that circ-MFN2 was resistant to the digestion of RNase R compared to linear MFN2, suggesting that circ-MFN2 indeed had a circular structure (Figures 1G,H).

### Knockdown of circ-MFN2 Inhibited the Proliferation, Metastasis, and Radioresistance of CRC

**FIGURE 2:**
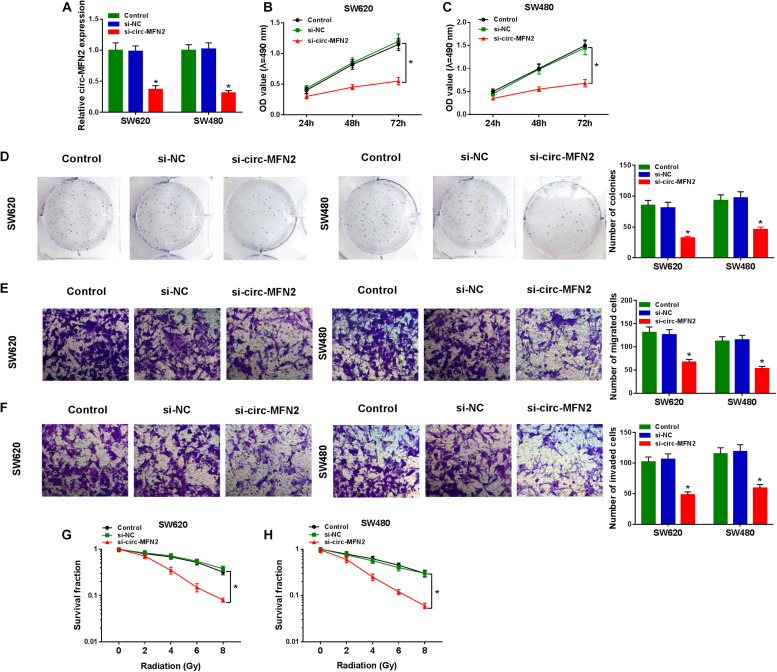
Circ-MFN2 knockdown inhibited CRC progression. SW620 and SW480 cells were transfected with si-NC or si-circ-MFN2, and non-transfected cells were used as control. **(A)** The expression of circ-MFN2 was detected by qRT-PCR to evaluate transfection efficiency. **(B,C)** MTT assay was used to measure the viability of cells. **(D)** The number of colonies was determined using colony formation assay. **(E,F)** Transwell assay was used to assess the migration and invasion of cells. **(G,H)** The survival fraction of cells was examined using colony formation assay. *P < 0.05.

For investigating the role of circ-MFN2 in CRC, we knocked down circ-MFN2 expression using si-circ-MFN2. Through detecting the expression of circ-MFN2 and linear MFN2, we confirmed that si-circ-MFN2 could effectively reduce the expression of circ-MFN2 (Figure 2A), while not affect the mRNA and protein expression levels of linear MFN2 (Supplementary Figures 1A,B). MTT assay and colony formation assay results indicated that circ-MFN2 knockdown could suppress the viabilities and the colony numbers of SW620 and SW480 cells, suggesting that the proliferation of CRC was hindered by circ-MFN2 silencing (Figures 2B–D). Moreover, the results of transwell assay showed that silenced circ-MFN2 also repressed the numbers of migrated and invaded SW620 and SW480 cells (Figures 2E,F). In addition, the survival fractions of SW620 and SW480 cells were inhibited by circ-MFN2 knockdown, indicating that silenced circ-MFN2 restrained the radioresistance of CRC cells (Figures 2G,H).

### Circ-MFN2 Silencing Reduced CRC Tumor Volume and Weight *in vivo*

**FIGURE 3:**
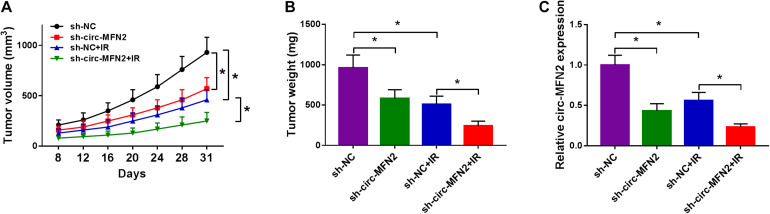
Circ-MFN2 silencing reduced CRC tumor growth *in vivo*. SW480 cells transfected with sh-NC or sh-circ-MFN2 were injected into nude mice. 6 Gy radiation was given after 8 days, and then radiation was given every 4 days until 31 days. The tumor volume **(A)** and tumor weight **(B)** were measured in mice. **(C)** QRT-PCR was used to detect the expression of circ-MFN2 in tumors. *P < 0.05.

To further confirm the role of circ-MFN2 in CRC, we constructed CRC tumor xenograft models. Through detecting the tumor volume and tumor weight, we found that the absence of circ-MFN2 significantly reduced the tumor volume and tumor weight of CRC, and markedly enhanced the sensitivity of CRC tumors to radiation (Figures 3A,B). Meanwhile, the detection results of circ-MFN2 expression in tumors suggested that circ-MFN2 was indeed inhibited in the sh-circ-MFN2 group, and that radiation could significantly hinder the expression of circ-MFN2 (Figure 3C). All data indicated that circ-MFN2 silencing could restrain the tumor growth of CRC.

### Circ-MFN2 Directly Interacted With miR-574-3p

**FIGURE 4:**
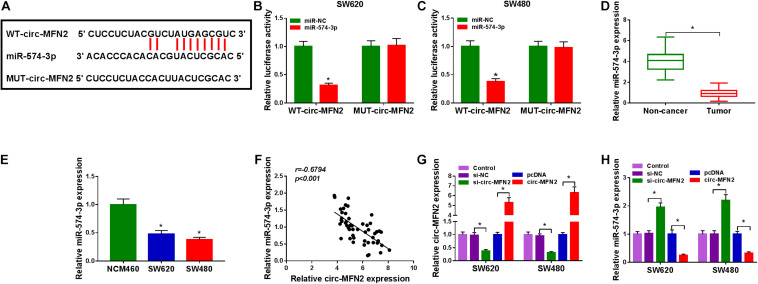
Circ-MFN2 directly interacted with miR-574-3p. **(A)** The predicted and mutated binding sites between circ-MFN2 and miR-574-3p were shown. **(B,C)** Dual-luciferase reporter assay was used to verify the interaction between circ-MFN2 and miR-574-3p. **(D)** The expression of miR-574-3p in CRC tumor tissues and non-cancer tissues was measured using qRT-PCR. **(E)** QRT-PCR was used to detect the miR-574-3p expression in CRC cell lines (SW620 and SW480) and NCM460 cells. **(F)** Pearson correlation analysis was used to assess the correlation between circ-MFN2 and miR-574-3p. **(G)** The transfection efficiencies of si-circ-MFN2 and pcDNA circ-MFN2 overexpression plasmid were confirmed using qRT-PCR. **(H)** QRT-PCR was employed to detect the expression of miR-574-3p to assess the effect of circ-MFN2 on miR-574-3p expression. *P < 0.05.

To perfect the mechanism of circ-MFN2, the StarBase v2.0 tool was used for predicting the targeted miRNAs of circ-MFN2. We found that miR-574-3p had binding sites with circ-MFN2 (Figure 4A). Based on this, we constructed the WT-circ-MFN2 and MUT-circ-MFN2 reporter vectors to carry out dual-luciferase reporter assay. The results showed that the luciferase activity of WT-circ-MFN2 vector was inhibited by miR-574-3p overexpression. However, the luciferase activity of MUT-circ-MFN2 vector had no statistical changes (Figures 4B,C). In CRC tumor tissues and cell lines, we discovered that miR-574-3p was lowly expressed compared with that in negative controls (Figures 4D,E). Moreover, the expression of miR-574-3p was negatively correlated with the expression of circ-MFN2 in CRC tissues (Figure 4F). To further determine the role of circ-MFN2 in the regulation of miR-574-3p, si-circ-MFN2 and pcDNA circ-MFN2 overexpression plasmid were transfected into SW620 and SW480 cells. The significant inhibition of circ-MFN2 expression by si-circ-MFN2 and the significant promotion of circ-MFN2 expression by pcDNA circ-MFN2 overexpression plasmid confirmed that the transfection of both was successful (Figure 4G). QRT-PCR results indicated that the expression of miR-574-3p could be enhanced by circ-MFN2 silencing, while suppressed by circ-MFN2 overexpression (Figure 4H). These data showed that circ-MFN2 could sponge miR-574-3p in CRC.

### MiR-574-3p Inhibitor Reversed the Negative Regulation of circ-MFN2 Silencing on CRC Progression

**FIGURE 5:**
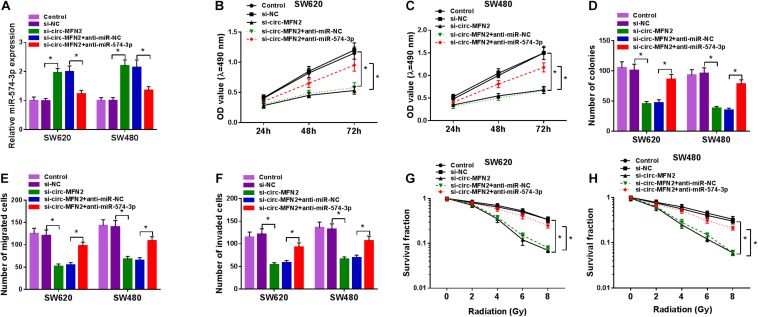
The regulation of circ-MFN2 silencing and miR-574-3p inhibitor on CRC progression. SW620 and SW480 cells were transfected with si-NC, si-circ-MFN2, si-circ-MFN2 + anti-miR-NC or si-circ-MFN2 + anti-miR-574-3p, and non-transfected cells were used as control. **(A)** The expression of miR-574-3p was measured by qRT-PCR to evaluate transfection efficiency. **(B,C)** The viability of cells was detected using MTT assay. **(D)** The number of colonies was assessed using colony formation assay. **(E,F)** The migration and invasion of cells were examined by transwell assay. **(G,H)** Colony formation assay was performed to detect the survival fraction of cells. *P < 0.05.

Subsequently, we co-transfected with si-circ-MFN2 and anti-miR-574-3p into SW620 and SW480 cells to determine whether circ-MFN2 regulated CRC progression by targeting miR-574-3p. As presented in Figure 5A, compared with the control group, the significantly decreased expression of miR-574-3p in the si-circ-MFN2 + anti-miR-574-3p group confirmed that anti-miR-574-3p had a good inhibitory effect on miR-574-3p expression. Then, we measured the proliferation, metastasis and radioresistance of CRC cells. The results of MTT assay and colony formation assay indicated that the inhibition effect of circ-MFN2 knockdown on the viabilities and the colony numbers of SW620 and SW480 cells could be reversed by miR-574-3p inhibitor (Figures 5B–D). Furthermore, miR-574-3p inhibitor also inverted the suppressive effect of circ-MFN2 silencing on the migration and invasion of SW620 and SW480 cells (Figures 5E,F). Also, by measuring the survival fractions of SW620 and SW480 cells, we uncovered that the repressing effect of circ-MFN2 knockdown on the radioresistance of CRC cells could be reversed by miR-574-3p inhibitor (Figures 5G,H). Therefore, our data suggested that circ-MFN2 regulated CRC progression by sponging miR-574-3p.

### MiR-574-3p Could Target IGF1R

**FIGURE 6:**
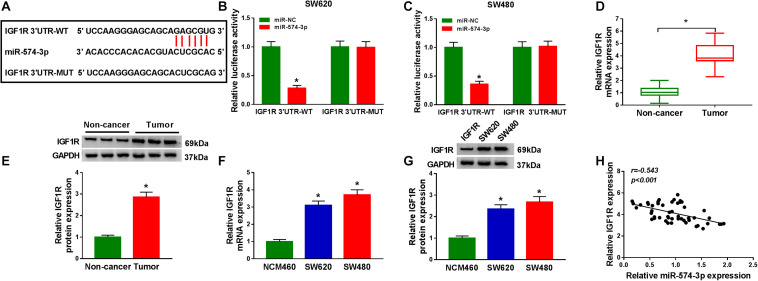
MiR-574-3p could target IGF1R. **(A)** The sequences of IGF1R 3′UTR-WT and IGF1R 3′UTR-MUT were shown. **(B,C)** The interaction between IGF1R 3′UTR and miR-574-3p was confirmed using dual-luciferase reporter assay. **(D,E)** The mRNA and protein levels of IGF1R in CRC tumor tissues and non-cancer tissues were measured using qRT-PCR and WB analysis. **(F,G)** QRT-PCR and WB analysis were used to detect the mRNA and protein levels of IGF1R in CRC cell lines (SW620 and SW480) and NCM460 cells. **(H)** The correlation between IGF1R and miR-574-3p was analyzed using Pearson correlation analysis. *P < 0.05.

In order to determine the targeted genes of miR-574-3p, the StarBase v2.0 tool was used to conduct bioinformatics analysis. IGF1R 3′UTR was found to have binding sites of miR-574-3p, as shown in Figure 6A. The results of dual-luciferase reporter assay revealed that miR-574-3p only could inhibit the luciferase activity of IGF1R 3′UTR-WT vector without affect the luciferase activity of IGF1R 3′UTR-MUT vector (Figures 6B,C). Furthermore, using the qRT-PCR and WB analysis, we discovered that the mRNA and protein levels of IGF1R were remarkably upregulated in CRC tissues and cell lines compared with that in non-cancer tissues and NCM460 cells, respectively (Figures 6D–G). In addition, correlation analysis results indicated that there had a negative correlation between IGF1R and miR-574-3p expression in CRC tissues (Figure 6H). These results showed that IGF1R was a target of miR-574-3p.

### IGF1R Overexpression Reversed the Inhibition Effect of miR-574-3p on CRC Progression

**FIGURE 7:**
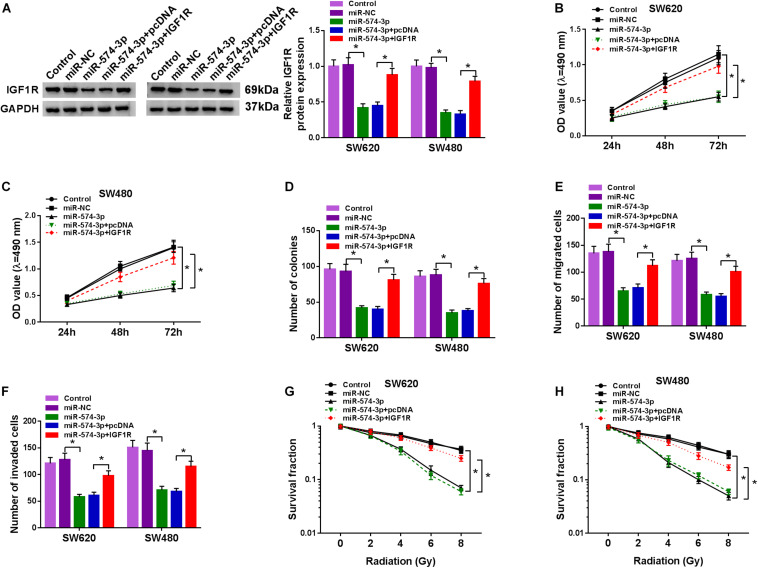
The regulation of miR-574-3p mimic and IGF1R overexpression on CRC progression. SW620 and SW480 cells were transfected with miR-NC, miR-574-3p, miR-574-3p + pcDNA or miR-574-3p + IGF1R, and non-transfected cells were used as control. **(A)** The protein level of IGF1R was measured by WB analysis to evaluate transfection efficiency. **(B,C)** The viability of cells was assessed using MTT assay. **(D)** Colony formation assay was determined to evaluate the number of colonies. **(E,F)** The migration and invasion of cells were detected using transwell assay. **(G,H)** The survival fraction of cells was examined using colony formation assay. *P < 0.05.

To further determine that miR-574-3p regulated CRC progression by targeting IGF1R, we co-transfected with miR-574-3p mimic and pcDNA IGF1R overexpression plasmid into SW620 and SW480 cells. Through detecting the protein expression of IGF1R, we found that miR-574-3p overexpression could inhibit IGF1R expression, while this effect could be reversed by the addition of pcDNA IGF1R overexpression plasmid, which confirmed the successful transfection of both (Figure 7A). The detection results of the viabilities and colony numbers of SW620 and SW480 cells suggested that miR-574-3p overexpression suppressed CRC cell proliferation, and this effect could be reversed by IGF1R overexpression (Figures 7B–D). Moreover, the suppressive effect of miR-574-3p mimic on the migration and invasion of SW620 and SW480 cells also was reversed by IGF1R overexpression (Figures 7E,F). In addition, miR-574-3p mimic reduced the survival fractions of SW620 and SW480 cells, while the addition of IGF1R overexpression plasmid could recover the inhibition effect of miR-574-3p mimic on the radioresistance of CRC cells (Figures 7G,H). Hence, our results presented that miR-574-3p hindered CRC progression via suppressing IGF1R.

### Circ-MFN2 Sponged miR-574-3p to Positive Regulate IGF1R Expression

**FIGURE 8:**
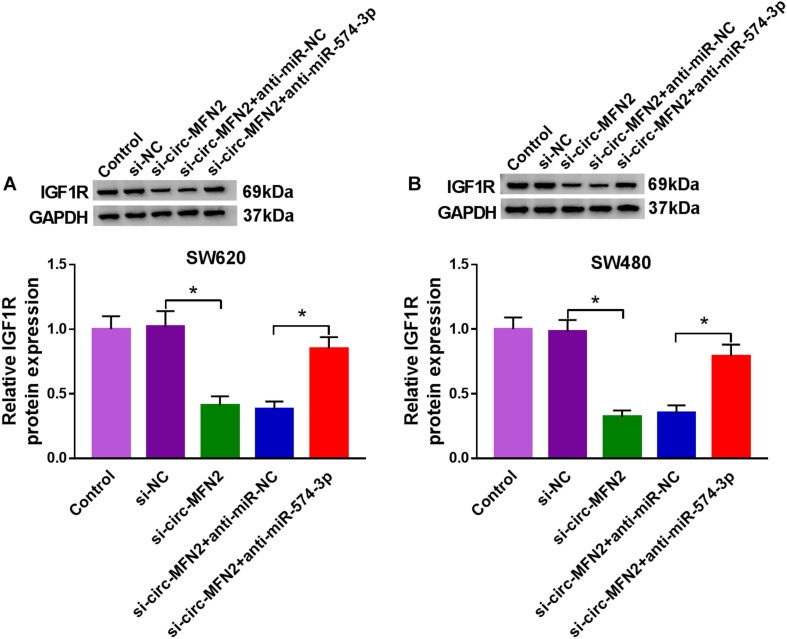
Circ-MFN2 and miR-574-3p regulated IGF1R expression. SW620 and SW480 cells were transfected with si-NC, si-circ-MFN2, si-circ-MFN2 + anti-miR-NC or si-circ-MFN2 + anti-miR-574-3p, and non-transfected cells were used as control. The protein level of IGF1R in SW620 **(A)** and SW480 **(B)** cells was measured using WB analysis. *P < 0.05.

To confirm the regulation of circ-MFN2 on IGF1R expression, we measured IGF1R expression in SW620 and SW480 cells co-transfected with si-circ-MFN2 and anti-miR-574-3p. WB analysis results revealed that circ-MFN2 knockdown repressed the expression of IGF1R in SW620 and SW480 cells, and this effect could be reversed by miR-574-3p inhibitor (Figures 8A,B). Therefore, we confirmed that circ-MFN2 positively regulated IGF1R expression by targeting miR-574-3p.

## Discussion

Recent investigations supported the role of circRNAs in human cancer development. The carcinogenic or suppressive effect of circRNA provides evidence for the potential application of circRNA in cancer diagnosis and treatment (Cui et al., 2020; Tang and Hann, 2020). In this study, we focused on exploring the function of circ-MFN2 in CRC. Our results revealed that circ-MFN2 had increased expression in CRC, especially in the tissues of advanced CRC patients and those with metastasis. In view of the high expression of circ-MFN2, we silenced its expression in CRC. Our study discovered that circ-MFN2 silencing restrained the proliferation, metastasis and radioresistance of CRC cells *in vitro* and inhibited CRC tumor growth *in vivo*. These suggested that circ-MFN2 might have a pro-cancer role in CRC, which was similar with the function of circ-MFN2 in other cancers (Deng et al., 2018; Liu et al., 2018; Li and Liu, 2020).

CircRNA can act as a miRNA sponge to mediate downstream target expression (Thomson and Dinger, 2016; Witkos et al., 2018). To clarify the mechanism of circ-MFN2, we performed the bioinformatics analysis. The results showed that circ-MFN2 could sponge miR-574-3p. MiR-574-3p is often underexpressed in human cancers and is considered to be a tumor suppressor involved in the regulation of cancer progression (Xu et al., 2016). Some research indicated that miR-574-3p inhibited the proliferation and metastasis of esophageal cancer and ovarian cancer (Zheng et al., 2019; Jin et al., 2020). More importantly, Wang et al. reported that miR-574-3p could suppress the EMT and cisplatin resistance of gastric carcinoma cells (Wang M. et al., 2019). In CRC, miR-574-3p was found to have an inhibitory effect on CRC cell proliferation, metastasis (Li et al., 2019). Consistent with these studies, our research showed that miR-574-3p was downregulated in CRC tissues compared with non-cancer tissues. The rescue experiments further confirmed that miR-574-3p was involved in the regulation of circ-MFN2 on CRC proliferation, metastasis and radioresistance. Further bioinformatics analysis predicted that miR-574-3p could target IGF1R.

IGF1R is a transmembrane receptor that belongs to the tyrosine kinase family (Chitnis et al., 2008). Existing research shows that IGF1R is abnormally expressed in many cancers, which is closely related to the development of malignant tumors (Salisbury and Tomblin, 2015). Therefore, molecular targeted therapy for IGF1R has also become an attractive cancer treatment direction (Tognon and Sorensen, 2012; Werner et al., 2019). In CRC, high expression of IGF1R was believed to enhance CRC radioresistance (Afshar et al., 2018), and its knockdown could inhibit the metastasis of CRC (Liu et al., 2017). Similar to the previous study, our results uncovered that IGF1R was upregulated in CRC. The reversal effect of IGF1R on miR-574-3p confirmed that IGF1R was the target of miR-574-3p and could participate in the regulation of CRC progression by miR-574-3p, which also showed that IGF1R could promote the proliferation, metastasis and radioresistance of CRC. In addition, we found that IGF1R expression was positively regulated by circ-MFN2 and negatively regulated by miR-574-3p, which perfected the existence of circ-MFN2/miR-574-3p/IGF1R axis in CRC.

In conclusion, our studies revealed that circ-MFN2 was an upregulated circRNA in CRC. Functionally, circ-MFN2 could facilitate the proliferation, metastasis and radioresistance of CRC by regulating the miR-574-3p/IGF1R axis, suggesting that circ-MFN2 might function as an oncogene in CRC. Our findings revealed the role and potential mechanism of circ-MFN2 in CRC progression for the first time, and provided new targets for the treatment of CRC.

## Data Availability Statement

The datasets used and/or analyzed during the current study are available from the corresponding author on reasonable request.

## Ethics Statement

The studies involving human participants were reviewed and approved by the Fourth Affiliated Hospital of Anhui Medical University. The patients/participants provided their written informed consent to participate in this study. The animal study was reviewed and approved by The Fourth Affiliated Hospital of Anhui Medical University.

## Author Contributions

DL designed the study. DL and SP analyzed the data. YL performed the experiments. DL and TG summarized the data and wrote the manuscript. All authors contributed to this study, read and approved the manuscript.

## Conflict of Interest

The authors declare that the research was conducted in the absence of any commercial or financial relationships that could be construed as a potential conflict of interest.

## Supplementary Material

Supplementary Figure 1 The expression of linear MFN2 under the treatment of si-circ-MFN2. SW620 and SW480 cells were transfected with si-NC or si-circ-MFN2, and non-transfected cells were used as control. The mRNA and protein expression levels of MFN2 were determined using qRT-PCR **(A)** and WB analysis **(B)**.
