# Supplementary figures and images for: Circ-MFN2 Positively Regulates the Proliferation, Metastasis, and Radioresistance of Colorectal Cancer by Regulating the miR-574-3p/IGF1R Signaling Axis
Source: Front Genet. 2021 May 19;12:671337. doi: 10.3389/fgene.2021.671337 (PMC8170135; doi:10.3389/fgene.2021.671337)

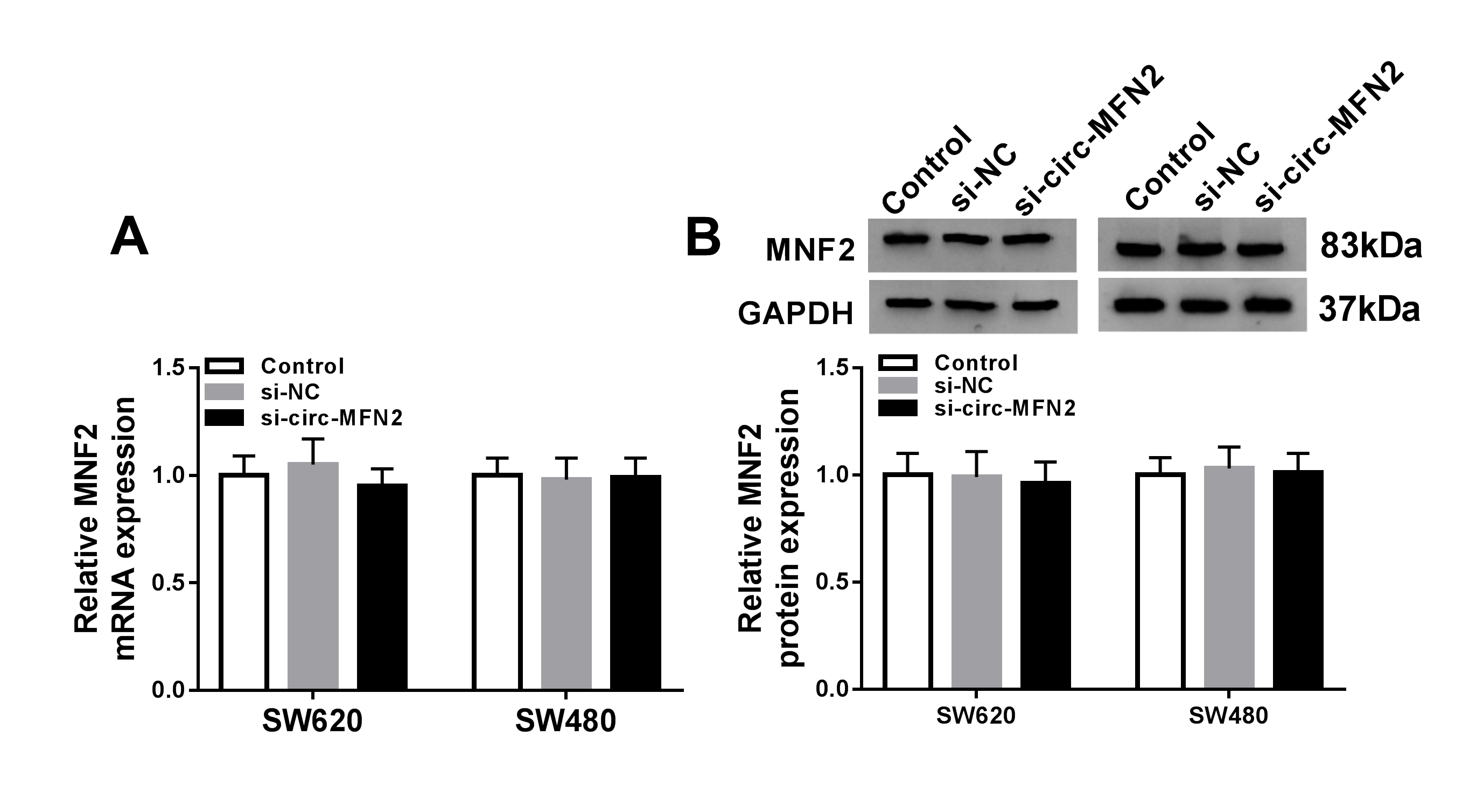

Supplement: Supplementary Figure 1 — The expression of linear MFN2 under the treatment of si-circ-MFN2. SW620 and SW480 cells were transfected with si-NC or si-circ-MFN2, and non-transfected cells were used as control. The mRNA and protein expression levels of MFN2 were determined using qRT-PCR (A) and WB analysis (B). [file Image_1.TIF]
